# A Dual-Color Fluorescence-Based Platform to Identify Selective Inhibitors of Akt Signaling

**DOI:** 10.1371/journal.pone.0001823

**Published:** 2008-03-19

**Authors:** Aranzazú Rosado, Fabian Zanella, Beatriz Garcia, Amancio Carnero, Wolfgang Link

**Affiliations:** Experimental Therapeutics Program, Centro Nacional de Investigaciones Oncologicas (CNIO), Madrid, Spain; National Institutes of Health, United States of America

## Abstract

**Background:**

Inhibition of Akt signaling is considered one of the most promising therapeutic strategies for many cancers. However, rational target-orientated approaches to cell based drug screens for anti-cancer agents have historically been compromised by the notorious absence of suitable control cells.

**Methodology/Principal Findings:**

In order to address this fundamental problem, we have developed BaFiso, a live-cell screening platform to identify specific inhibitors of this pathway. BaFiso relies on the co-culture of isogenic cell lines that have been engineered to sustain interleukin-3 independent survival of the parental Ba/F3 cells, and that are individually tagged with different fluorescent proteins. Whilst in the first of these two lines cell survival in the absence of IL-3 is dependent on the expression of activated Akt, the cells expressing constitutively-activated Stat5 signaling display IL-3 independent growth and survival in an Akt-independent manner. Small molecules can then be screened in these lines to identify inhibitors that rescue IL-3 dependence.

**Conclusions/Significance:**

BaFiso measures differential cell survival using multiparametric live cell imaging and permits selective inhibitors of Akt signaling to be identified. BaFiso is a platform technology suitable for the identification of small molecule inhibitors of IL-3 mediated survival signaling.

## Introduction

Cell-based screens have been widely used in drug discovery although historically, these assays are conducted using genetically diverse cell lines derived from human tumors [1], [2]. Since the complex intracellular signaling networks that drive cancer cell growth and survival have begun to be elucidated, a more rational approach to drug discovery has become feasible [3]. However, the implementation of target-orientated cell-based screens for anti-cancer drugs remains a challenge, both because of their reliance on defined genetic changes and because of the lack of proper control cells. To overcome this fundamental problem, we have developed a rational strategy for cell-based drug discovery that is based on the convenience and flexibility of the Ba/F3 cell system, an immortalized IL-3-dependent pro-B lymphoblastic cell line [4]. IL-3 supports the growth and survival of Ba/F3 cells through the activation of distinct signaling pathways. Upon binding to its cognate receptor IL-3 activates the Janus kinase signal transduction and transcriptional activation pathways (JAK/STAT) to induce Bcl-x_L_ [5]. Similarly, IL-3 activation of the PI3K/Akt pathway is involved in inhibiting the intrinsic apoptotic machinery in Ba/F3 cells [6]–[8].

Overexpression of several constitutively active signaling molecules abrogates the dependence of these cells on IL-3 [9]. Hence, we generated isogenic cell lines derived from Ba/F3 (BaFiso) in which IL-3 independent survival is sustained by independent signaling events. Each of these isogenic lines was genetically labeled with a fluorescent reporter and thus, the ratio of two spectrally distinct cell populations could be used as primary endpoint of the system to monitor pathway-specific cytotoxicity. Accordingly compounds can be screened in co-cultures of these lines and the change in the relative cell number of the two lines readily and rapidly measured to identify those molecules that specifically interact with one of the signaling pathways. In this instance, BaFiso has been designed as a live-cell system suitable to identify specific inhibitors of Akt signaling.

## Results

### Tagging isogenic Ba/F3 cells individually with two different chromophores

**Figure 1:**
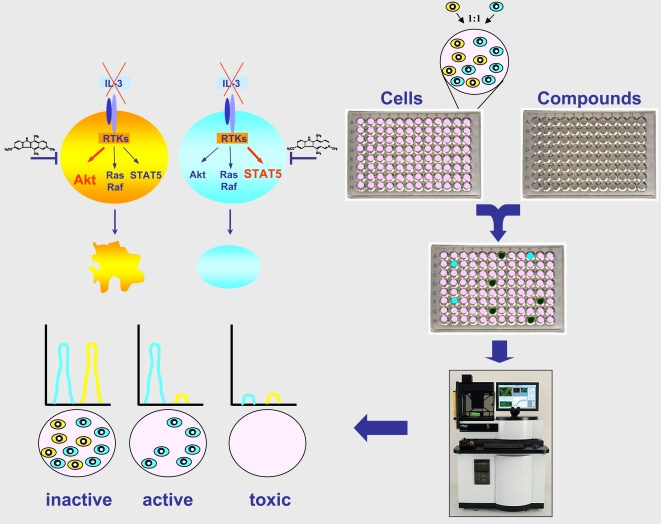
Schematic overview of the BaFiso assay system. BaFiso consists of paired isogenic cell lines that have been engineered to acquire IL-3 autonomous growth through constitutive activation of Akt or Stat5 signaling. The two cell lines to be compared are individually tagged with either yellow or cyan fluorescent proteins. Equal numbers of yellow and cyan cells were co-cultured, treated with compounds and the change in the relative cell number was calculated on the basis of the distinct fluorescent proteins measured. Our strategy aims to identify lead compounds that specifically kill test cells with activated Akt signaling (yellow cells) and that spare the otherwise isogenic control cells (cyan cells).

**Figure 2:**
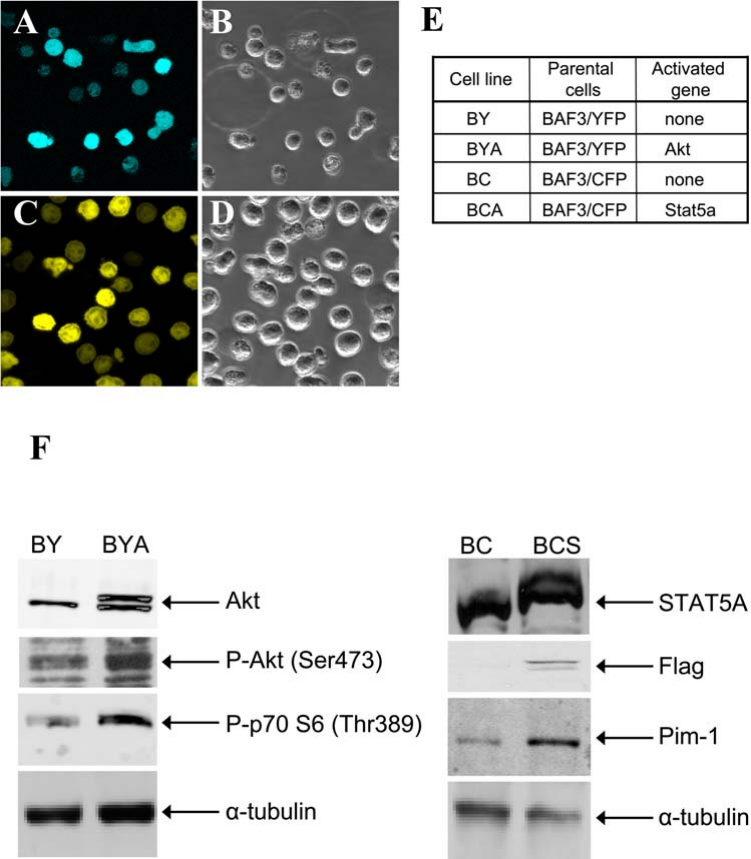
The generation of BaFiso cell lines. Ba/F3 cells were transduced with retroviral supernatant carrying pBabePuro-EYFP or pBabePuro-ECFP. Cell clones were established and sorted in a fluorescence activated cell sorter (FACS) to generate lines homogeneously expressing the corresponding fluorescent tags. (A) and (C), viable Ba/F3 cells show robust and homogeneous expression of the respective fluorescent protein. (B) and (D), corresponding light field views. (E), Generation of stable BaFiso cell lines. Clonal Ba/F3 cells stably expressing EYFP (BY) or ECFP (BC) were used to generate stable BaFiso cell lines that co-express yellow fluorescence and myr-Akt (BYA), or cyan fluorescence and STAT5A1*6 (BCS). Cell clones were established and analyzed. (F), Analysis of transgene expression and downstream activation of the corresponding signaling pathways by western blotting. Antibodies against total Akt, Stat5a, Flag phospho-Akt (Ser473), phospho-p70 S6 (Thr389) and Pim-1 were used and the signals normalized to the respective α-tubulin levels.

The BaFiso system is a dual fluorescence cell-based screening system in which compounds can be readily monitored thanks to the stable expression of yellow or cyan fluorescent proteins that individually tag each of the isogenic cell lines (Fig. 1). To introduce the genes encoding the different fluorescent proteins into Ba/F3 cells, retroviral supernatants were generated by transfection of LinX packaging cells. Through clonal propagation, we were able to establish Ba/F3 cell lines that robustly and homogeneously expressed ECFP (Fig. 2A and B) or EYFP (Fig. 2C and D). Stable transfectants of these proteins were FACS-sorted to ensure that they expressed similar levels of the fluorescent reporter protein.

### Generation of double stable Ba/F3 cell lines

The strategy described here is based on paired isogenic cell lines whose survival in the absence of IL-3 is sustained by the activation of independent signaling pathways. Several signaling pathways have been implicated in IL-3-mediated survival, including those involving Akt and Stat5 [10], [11]. In order to introduce constitutively active forms of these genes into our dual fluorescence cell-based system, we used retroviral constructs carrying a myristoylated derivate of Akt and STAT5A1*6, which contains two activating amino acid substitutions [11]. The yellow labeled Ba/F3 cells were used to generate Akt-dependent reporter cells whereas the cyan tagged cells were used to establish PI3K/Akt independent reporter cells. The retroviral supernatants of LinX packaging cells were employed to transduce Ba/F3/EYFP cells (BY) with myr-Akt and Ba/F3/ECFP cells (BC) with STAT5A1*6 (Fig. 2E). Stably expressing cell clones were selected and the expression of the transgenes was confirmed by western blot analysis (Fig. 2F). The level of Akt expression was monitored using an antibody that recognizes Akt irrespective of its phosphorylation state. Akt migrates as a single band with an apparent molecular weight of 60 kDa, although a larger protein was also identified in immunoblots of Akt from lysates of BYA cells. This additional form can be explained by the difference in size produced by the myristoylation signal present in the constitutive active form of Akt used to generate the BYA cell line. Despite the Stat5a protein present in the parental BC cells, ectopic expression of the constitutively active form of Stat5a in BCS cells could be unequivocally demonstrated in western blots probed with an antibody directed against the Flag-tag. Indeed, STAT5A1*6 expression also increased the total Stat5a protein level in BCS cells as shown by immunoblotting using an antibody recognizing Stat5.

To examine whether PI3K/Akt or Stat5 signaling is indeed activated in the stable BYA or BCS cells respectively, we analyzed downstream elements in these two pathways. Phosphorylation of Akt (Ser473) has been widely used as a read out of activation of the PI3K pathway. When we compared the level of Akt phosphorylation in lysates of BY and BYA cells cultured in the presence of IL-3, there was dramatic increase in Ser473 phosphorylation of Akt in BYA cells, reflecting the activity of this pathway. To investigate whether the activation of Akt in BYA cells had an impact on downstream events, we analyzed the Thr389 phosphorylation of the linker domain of the p70 S6 kinase that is constitutively activated upon overexpression of a gag fusion of Akt [12]. There was a significant increase in the intensity of the band corresponding to p70 S6 kinase (Thr389) in BYA cells when compared to BY control cells. On the other hand, the expression of the known STAT5 target gene, pim-1, was upregulated upon expression of constitutive activated Stat5a, consistent with previous studies [13].

### Ectopic expression of activated Akt and Stat5a confers IL-3 independence

**Figure 3:**
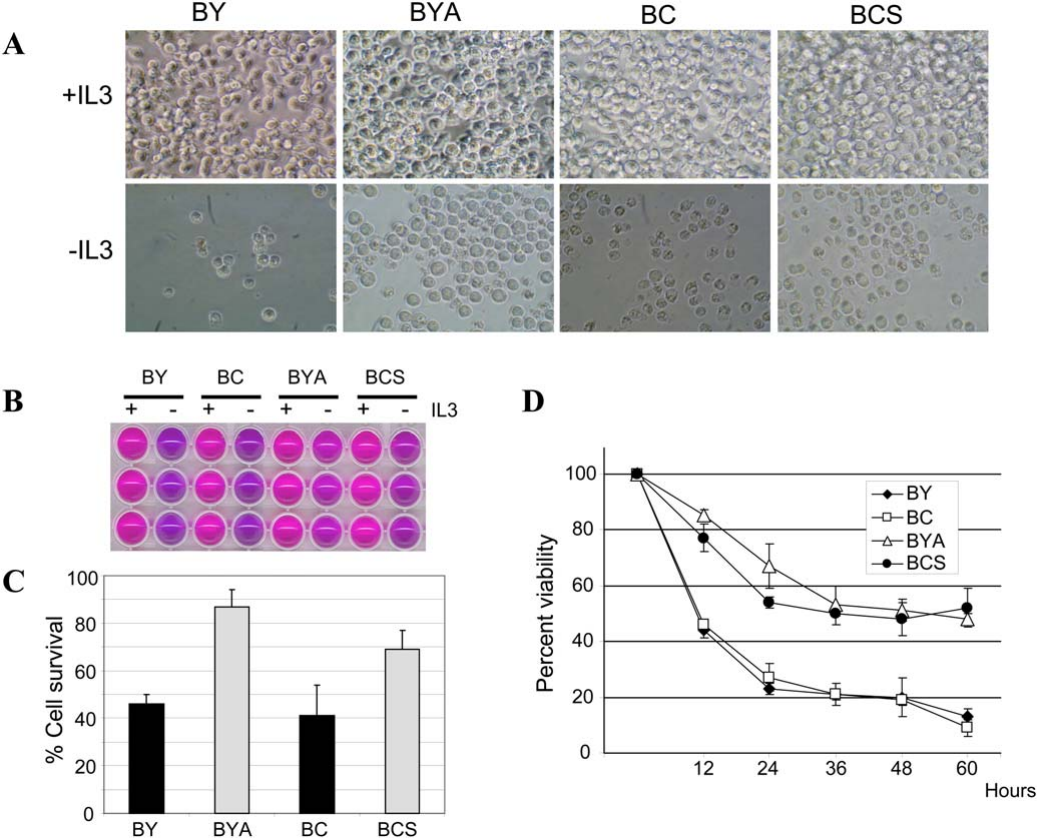
The viability of BaFiso cell lines in the absence of IL-3. (A) Parental Ba/F3 derived BY and BC cells, and the BaFiso cell lines BYA and BCS were maintained in the presence or absence of IL-3 (+IL3 and −IL3). Photos were taken 24 h after transferring the cells to medium without IL-3 or in the presence of 3 ng/ml of the recombinant cytokine. (B) Measurement of cell viability using the Alamar blue assay. Alamar blue fluoresces and changes color in response to chemical reduction, and the extent of the conversion is a reflection of cell viability. Metabolic conversion of Alamar blue to its reduced, pink derivative upon cytokine-deprivation (−IL-3) or in its presence (+IL-3). (C), Bar graph showing the results of Alamar Blue cell viability assay. Maximal absorbance of the reduced and oxidized forms of AlamarBlue™, 570 and 600 nm was measured using Victor 1420 multilabel counter 24 h after IL-3 withdrawal. The percentage of cell survival was calculated compared with control cells in the presence of 3 ng/ml of IL3. The data represents three independent experiments performed in triplicate samples. (D) Time course of cell viability upon IL-3 withdrawal. Cells were washed twice in PBS and seeded in media lacking IL-3. Viability was assessed at 12 hour intervals by trypan blue exclusions followed by cell countings. Black rhombs and open squares represent percentage viability of BY and BC cells, respectively. Open triangles and black circles represent percentage viability of BYA and BCS cells, respectively. Data are presented as mean±SD from three independent experiments.

Consistent with previous reports, expression of constitutively active mutants of Akt and Stat5a provide signals for cytokine-independent survival of Ba/F3 cells [9], [11]. The increased resistance to IL-3 withdrawal of the BYA and BCS cell lines when compared to the parental BY and BC cell lines was confirmed by morphological assessment. Parental BY and BC cells were cultured in the presence or absence of IL-3 and the degree of cell death was assessed after 24 hours by microscopic examination (Fig. 3A). The number of cells with an apoptotic phenotype increased significantly after IL-3 withdrawal in the cultures. The effect of the constitutive activation of Akt or Stat5 signaling was examined when IL-3 was withdrawn from representative BYA and BCS cell clones. As such, the capacity of the constitutively active forms of the signaling molecules Akt and Stat5a to impede apoptosis was evident and accordingly, cell death was dramatically reduced in Ba/F3 cells ectopically expressing myr-Akt or STAT5A1*6, even in the absence of IL-3 (Fig. 3A). We also determined the metabolic activity as a measure of cell viability using the alamar blue assay, in which a redox indicator changes color from blue to pink depending on metabolic status of the cells (Fig. 3B). The activity of myr-Akt in BYA cells was significantly higher in the absence of IL-3 than that of the parental cells. Similarly, STAT5A1*6 also maintained the activity of BCS cells albeit to slightly lesser degree (Fig. 3C). We examined the time course of cell viability following IL-3 withdrawal (Fig. 3D) and 24 hours after IL-3 deprivation, approximately 60% of the BYA or BCS cells remained viable compared to approximately 25% of the parental BY and BC cell lines. The viability of BY and BC cells further diminished after 60 hours of IL-3 starvation to 13% and 9%, respectively. In contrast, the viability of BYA and BCS cells remained around 50% after 60 hours in the absence of IL-3.

### The protection from IL-3 withdrawal afforded by enhanced Stat5 signaling is independent of Akt activity

**Figure 4:**
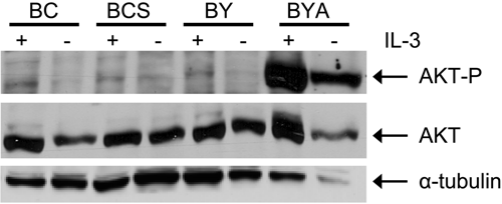
The analysis of Akt phosphorylation in BaFiso cell lines. Immunoblot analysis of total lysates from the Ba/F3 derived cell lines BY, BC, BYA and BCS. Cells were seeded, grown to 80% confluence and starved for 12 h in IL-3 free medium (−IL-3) or maintained in medium containing 3 ng/ml of the recombinant cytokine (+IL-3). Relevant proteins are indicated by arrows in the blot from a representative experiment.

The capacity to monitor pathway-specific cytotoxicity in our assay is based on the use of isogenic control cells that confer survival in the absence of IL-3 in an Akt-independent manner. Since Akt is one of the major downstream targets of PI3K signaling, its phosphorylation status is commonly used to monitor the activity of the PI3K/Akt pathway. We analyzed the impact of ectopic expression of myr-Akt and STAT5A1*6 on Akt activation using an antibody that specifically recognizes Ser473 phosphorylated Akt (Fig. 4). The intensity of Akt phosphorylation was compared to the overall expression of Akt and α-tubulin using specific antibodies. Parental BY and BC cells possess relatively low basal levels of Akt phosphorylation which further decreased upon withdrawal of IL-3. Ectopic constitutively active Stat5a expression had no significant impact on the phosphorylation of Akt in the presence or absence of IL-3, indicating that the enhanced survival of BCS cells triggered by STAT5A1*6 upon IL-3 starvation is independent of Akt signaling. Consistent with previous studies, overexpression of myr-Akt dramatically augmented Ser473 phosphorylation [14] and high levels of Akt phosphorylation were still detected in the complete absence of IL-3 (Fig. 4).

In conclusion, these results show that we have generated stable Ba/F3-derived cell lines in which the inhibition of the intrinsic apoptotic machinery is mediated by ectopic expression of constitutively active mutants of Akt or Stat5a. Since the abrogation of IL-3 dependence occurred through the activation of independent signaling pathways, these cell lines can be used together as paired isogenic test and control cells to identify pathway specific inhibitors.

### Detection of selective toxicity associated with activated Akt signaling

The BaFiso assay was set up in 96-well plates and with an automated workflow [15]. Equal numbers of BYA and BCS cells were mixed and seeded at a density of 20,000 cells per well using multidrop dispenser. All liquid handling for treatment and staining was carried out by a robotic workstation and the BD Pathway 855 cell imaging platform was used for automated image acquisition. In order to test the sensitivity and the capacity to detect EYFP and ECFP separately using BD Pathway 855 bioimager, the co-cultured cells were photographed for the two fluorochromes sequentially and the images superimposed. In order to avoid ECFP bleeding into the EYFP emission channel, a special filter set was used that clearly separates the two fluorochromes. A third fluorochrome, the far red/infrared fluorescent cell-permeant DNA probe, DRAQ5, was employed to perform automated segmentation of cell nuclei. An image algorithm was applied to segment the cell nucleus based on local thresholds. The ratios of the cyan and yellow fluorescence signals were determined by dividing the number of ECFP positive cells by the number of EYFP positive cells in each well.

As a proof of principle, we sought to determine how a panel of commercially available agents of known mechanism of action would behave in the BaFiso screen. The test compounds included: the DNA-damaging chemotherapeutic compound cisplatin; the modulator of membrane lipid structure Minerval; the Akt inhibitor 10-(4′-(N-diethylamino)butyl)-2-chlorophenoxazine (Akt Inhibitor X); the protein tyrosine kinase inhibitor Genistein; the inhibitor of nuclear export Leptomycin B; the broad protein kinase inhibitor Staurosporine; the PDK1 inhibitor UCN-01; the Raf1 Kinase Inhibitor; the PI3K inhibitor LY294002; the topoisomerase II inhibitor Etoposide; and Lithium chloride, a GSK-3 inhibitor.

**Figure 5:**
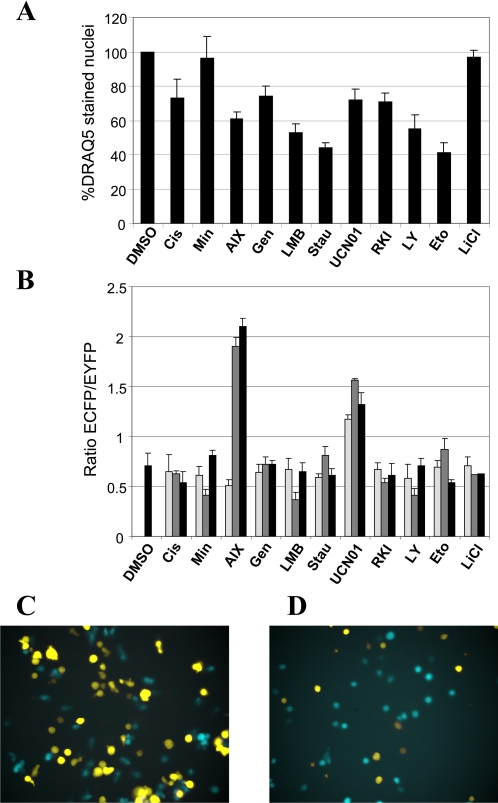
Validation of BaFiso assay using a panel of test compounds. (A) Analysis of the general toxicity of compound treatment. The total cell numbers in each well were determined by nuclear counterstain with the far-red fluorescent DNA probe DRAQ5. The number of DRAQ5-stained nuclei was determined after exposure to 30 µM Cisplatin (Cis), 100 µM Minerval (Min), 500 nM Akt Inhibitor X (AIX), 20 µM Genistein (Gen), 1 nM Leptomycin B (LMB), 20 nM Staurosporine (Stau), 1 µM UCN-01, 20 nM Raf1 Kinase Inhibitor (RKI), 20 µM LY294002 (LY), 10 µM Etoposide (Eto) and 1 mM lithium chloride (LiCl) for 12 hours and compared to vehicle treatment. (B) Equal numbers of BCS and BYA cells were co-cultured in IL-3-free medium. We exposed the paired BaFiso cell lines to 3 µM, 30 µM and 300 µM Cisplatin (Cis), 25 µM, 100 µM, 200 µM Minerval (Min), 50 nM, 500 nM, 5 µM Akt Inhibitor X (AIX), 200 nM, 20 µM, 50 µM Genistein (Gen), 0.5 nM, 1 nM, 4 nM Leptomycin B (LMB), 2 nM, 20 nM, 10 µM Staurosporine (Stau), 200 nM, 1 µM, 10 µM UCN-01; 5 nM, 20 nM, 200 nM Raf1 Kinase Inhibitor (RKI), 1 µM, 20 µM, 50 µM LY294002 (LY), 100 nM, 10 µM, 100 µM Etoposide (Eto) and 100 µM, 1 mM, 10 mM lithium chloride (LiCl), and Dimethyl sulfoxide (DMSO) as a negative control (striped bar). Three images specific for ECFP, EYFP or DRAQ5 from each well were acquired using BD Pathway Bioimager. The ECFP/EYFP ratio was determined by dividing the number of ECFP positive cells by the number of EYFP positive cells. Light, dark grey and black bars represent low, medium and high concentrations of the corresponding compounds, respectively. The data shown here represents three independent experiments. The average Z' value for BaFiso was 0.53. (C) Untreated, co-cultured BaFiso cells imaged before exposure to Akt Inhibitor X and (D) 12 h after treatment with 5 µM AIX.

A robotic workstation was used to prepare mother plates containing three different concentrations of these compounds. Co-cultured BaFiso BYA/BCS cells were exposed to equal volumes of the test compounds, resulting in a final concentration range greater than two orders of magnitude around the IC_50_ value for each compound. The final concentration of dimethyl sulfoxide was kept at 1% after addition of the compounds. Each plate contained several internal controls, including untreated wells and wells treated with different concentrations of DMSO or ethanol alone. The performance of the BaFiso system upon exposure to the panel of test compounds was measured in terms of the ECFP/EYFP ratio. The majority of the test compounds reduced the number of DRAQ5 positive cells (Fig. 5A) without affecting the ratio of cyan and yellow fluorescent signals (Fig. 5B), suggesting a non-selective cytotoxic effect on both BaFiso cell lines independent of the gene that has been engineered to sustain interleukin-3 independent survival of the cells. In contrast exposure to Minerval or LiCl did not affect the viability of the BaFiso cell lines (Fig. 5A) nor did it alter the ratio of the fluorescent signals (Fig. 5B). Most importantly, two compounds that are known to inhibit the kinase activity of Akt, UCN-01 and Akt Inhibitor X, [16], [17] selectively compromised the viability of the yellow tagged BYA cells thereby increasing the ratio of cyan to yellow fluorescent cells (Fig. 5B, C and D). In contrast, the broad spectrum PI3K isoform inhibitor LY294002 failed to affect the proportion of the fluorescent signals, indicating that the myristoylated form of Akt bypasses the requirement of PIP3-mediated membrane recruitment for its activity. Taken together, these data demonstrate that we have developed an image-based screening system that is capable of identifying specific inhibitors of the Akt pathway.

## Discussion

The most frequently used anti-cancer therapies were discovered on the basis of their anti-proliferative activity in functional cell assays but with no pre-existing knowledge of the mechanism of action. As a result none of the current drugs directly targets the molecular lesions responsible for malignant transformation and they are not selective. Indeed this lack of selectivity between cancer cells and normal cells is currently one of the main reasons for the failure of conventional chemotherapy. In recent years, our understanding of the genetics of human cancer has increased rapidly, enabling more rational approaches to drug discovery for anti-cancer therapies to be adopted. Accordingly, the present study set out to develop a rational cell-based drug discovery strategy, an approach that has historically been compromised by the lack of appropriate control cells [18].

With the objective of identifying lead compounds that specifically kill cells with activated Akt signaling and that spare control cells, we have combined the use of co-cultured isogenic cell lines with fluorescent technology. We introduced a myristoylated form of Akt which constitutively localizes to the plasma membrane, bypassing the requirement for PIP3 in Akt activation. This myr-Akt has been shown to constitutively inactivate pro-apoptotic downstream targets [14]. In order to generate Ba/F3 cells that survive in the absence of IL-3 independent of activated PI3K/Akt signaling, we transduced Ba/F3 cells with a retrovirus encoding STAT5A1*6, an activated mutant of STAT5. STAT5A1*6 has two amino acid substitutions and it is constitutively phosphorylated, localized in the cell nucleus and transcriptionally active in the absence of IL-3 [11]. In the BaFiso system presented here, the protective potential of myr-Akt is slightly greater than that provided by STAT5A1*6, which may be explained by the greater expression of myr-Akt. The design of the screen relies on the lack of relevant crosstalk between the pathways engineered to support IL-3 independent survival. Previous work has shown that the induced expression of bcl-xL and pim-1 promotes the IL-3-independent survival of Ba/F3 cells upon activation of STAT5 [13]. In contrast, studies in multiple cell lines suggest that Akt phosphorylates and inactivates proapoptotic proteins such as GSK-3β, Foxo3a and Bad in response to IL-3 [8], [19], [20]. We confirmed that the activation of Stat5 signaling in BCS cells did not increase Akt activity either in the presence or absence of IL-3.

Another common source of interference to be mitigated in multiplexed screening procedures is the bleed-through of fluorescence from one channel to the other. BaFiso allows simultaneous viewing of three different fluorescent signals and sharp separation of the emission signals from the cyan and yellow protein is achieved using a special filter set. We implemented BaFiso as an automated live-cell assay using a multidrop dispenser, a robotic workstation and a robotic cell imaging platform. We assessed the properties of this HTS co-culture assay using a panel of test compounds of known activity. The cytotoxicity of the test compounds was monitored by quantifying the DRAQ5 labelled cells and all compounds tested except LiCl and Minerval reduced the viability of Ba/F3 cells. The fact that only two compounds known to selectively interfere with Akt signaling, Akt inhibitor X and UCN-01, reduced the number of yellow tagged BYA cells demonstrates the specificity of the BaFiso system. The Akt inhibitor X is a N-substituted phenoxazine that inhibits the activity of Akt even in the absence of its pleckstrin homology domain and it has been suggested that it may bind in the ATP binding site [17]. In contrast, UCN-01 has been reported to inhibit several kinases including PDK1, a key regulator of Akt activity [16]. Interestingly, staurosporine that differs from UCN-01 only by the absence of a hydroxy group on the lactam ring failed to change the ratio of the BaFiso cell lines. A specificity analysis against a kinase panel revealed different patterns of inhibition for UCN-01 with respect to staurosporine [16]. It remains to be determined if these differences in specificity could account for the different behaviour observed for these two compounds in the BaFiso assay.

The BaFiso screening design presented here offers some major advantages over traditional *in vitro* biochemical assays or more classical cellular assays. Co-culture and simultaneous testing of the paired isogenic cell lines in this assay provides an internal control and eliminates errors resulting from separate assessments. BaFiso is an image based high throughput assay that enables compound that produce artefacts and cytotoxicity to be identified on a single cell basis. Live cell imaging of the BaFiso cell lines permits the repeated monitoring of the same cells over the timecourse of an experiment, leading to a more accurate assessment that minimizes the variability in cell numbers between wells. Finally, the dual fluorescence co-culture system used in BaFiso is adaptable to any gene or pathway that can support IL-3 independent survival of Ba/F3 cells.

## Methods

### Expression Vectors and Reagents

The enhanced fluorescent protein vectors (pECFP-C1 and pEYFP-C1) were purchased from Clontech. The cDNAs encoding ECFP and EYFP were subcloned into the SnaBI sites of the pBABE-puro retroviral vector. The myr-Akt was kindly provided by Dr. Philip Tsichlis and we PCR amplified myr-Akt-HA using forward 5′–CGCGGATCCATGGGGAGCAGCAAGAGCAAGC–3′ and reverse 5′–ACGCGTCGACTCATCTAGAAGCGTAATCTGGAACC–3′ primers, before subcloning the BamHI and SalI digested PCR product into the corresponding restriction site of the retroviral vector pWZL-Blast. The Stat5A1*6-Flag construct was a kind gift from T. Nosaka (University of Tokyo). The nature of all constructs was confirmed by DNA sequencing.

All chemicals were purchased from commercial sources except UCN-01 which was kindly provided by NCI, Cisplatin which was provided by C. Navarro (Universidad Autónoma de Madrid, Spain), and Minerval which was generously provided by P. Escriba (University of the Balearic Islands, Palma de Mallorca, Spain). The Akt Inhibitor X, LY294002 and Raf1 Kinase Inhibitor were purchased from Calbiochem (San Diego, CA), Leptomycin B and Genistein were purchased from LC Laboratories (Woburn, MA, USA), Lithium chloride (LiCl), Etoposide and Staurosporine ware all purchased from Sigma-Aldrich (St. Louis, USA).

### Cell Culture

Murine pro-B Ba/F3 cells were obtained from the American Type Culture Collection (ATCC) and maintained in RPMI 1640 containing: 10% fetal calf serum; 2 mM L-glutamine; 50 µM 2-mercaptoethanol (Sigma); antibiotics and antimycotics (Gibco); and 3 ng/ml of recombinant murine IL-3 (R&D Systems, Minneapolis, MN, USA). LinXE ecotropic retrovirus producing cells [21] were grown in Dulbecco's modified Eagle's medium with glutamax supplemented with 10% fetal bovine serum (FBS), penicillin, streptomycin and fungizone (Gibco). Cell cultures were maintained in a humified incubator at 37°C with 5% CO_2_. To remove IL-3, the cells were washed twice in PBS at room temperature. Retroviral constructs were introduced into packaging cells by standard calcium phosphate transfection and retroviral-mediated gene transfer was performed as described previously [22]. After infection of Ba/F3 cells with retroviral supernatants containing either EYFP or ECFP, stable cell lines were selected in medium containing 2 µg/ml of puromycin for one week. In order to establish Ba/F3 cell lines homogeneously expressing EYFP or ECFP, we performed clonal propagation in Clona-cell TCS semi-solid culture medium (Stem Cell Technologies, Vancouver, Canada) containing 2 µg/ml puromycin according to the manufacturer's protocol. Ba/F3 cell clones stably expressing EYFP (BY cells) or ECFP (BC cells) were used as parental cells for the secondary stable infection with retroviral supernatants containing either myr-Akt or Stat5A1*6-Flag, respectively. Stable Ba/F3 cells co-expressing EYFP and myr-Akt (BYA cells) were selected with 0.8 mg/ml Neomycin and 1 µg/ml puromycin for 2 weeks. Stable Ba/F3 cells co-expressing ECFP and Stat5A1*6-Flag (BCS cells) were selected with 15 µg/ml Blasticidine and 1 µg/ml puromycin for 2 weeks. The generation of cell clones was performed as described above. Fluorescence-activated cell sorting (FACS) of EYFP or ECFP expressing cells was performed on a FACSAria (BD Biosciences, San Jose, CA, USA).

### Western Blot Analysis

Cells incubated under different conditions were washed twice with TBS prior to lysis in buffer containing: 50 mM Tris HCl, 150 mM NaCl, 1% NP-40, 2 mM Na_3_VO_4_, 100 mM NaF, 20 mM Na_4_P_2_O_7_, and protease inhibitor cocktail (Roche Molecular Biochemicals, Indianapolis, IN). Proteins were resolved on 10% SDS-PAGE, and transferred to PVDF membranes (Immobilon-P, Millipore). The membranes were incubated with the first antibody overnight at 4°C, washed and incubated with anti-mouse (1∶10000) or anti-rabbit (1∶5000) horseradish peroxidase conjugated antibodies. Immunoreactive proteins were visualized using the enhanced chemiluminescence (ECL) Western blotting detection system (Amersham Pharmacia Biotech) and Kodak-X-Omat LS film (Kodak). Antibodies against phospho-AKT (Ser473) and AKT were purchased from Cell Signaling (Beverly, MA), those against STAT-5 from (R&D Systems, Minneapolis, MN, USA), and the antibodies against α-tubulin and Flag were obtained from Sigma (St Louis, MO).

### Survival assay

Each cell line was individually seeded at 10^4^ cells per well in a 96 well plate, in the presence or absence of IL-3. AlamarBlue™ (Serotec, Oxford, UK) was added to the culture medium at a final concentration of 10% (v-v) and after 24 hours, absorbance was measured at the two different wavelengths of maximal absorbance of the reduced and oxidized forms of AlamarBlue™, 570 and 600 nm using Victor 1420 Multilabel Counter (Perkin-Elmer, Wellesley, USA). The percentage cell survival was calculated according to the manufacturers' instructions. Time course experiments of cell viability post IL-3 withdrawal were performed using trypan blue exclusion.

### BaFiso assay

Equal numbers of parental BC/BY cells or activated test cells BCS/BYA were mixed in culture medium deprived of IL-3 and seeded in 96-well black clear bottom microplates coated with Poly-D-Lysine (Becton Dickinson Biosciences, San Jose, California, USA) at a density of 20,000 cells per well using Titan Multidrop 384 automatic dispenser (Titertek Instruments, Inc., Huntsville, AL). The final volume of the cell suspension was 200 µl in each well. After incubation at 37°C with 5% CO_2_ for 1 hour the far-red fluorescent cell-permeable DNA probe, DRAQ5™ (Biostatus Ltd, Leicestershire, UK) was added at a final concentration of 5 µM to all wells 15 minutes prior to obtaining the first images. Then, 2 µl of each test compound or vehicle was transferred from the mother plates to the assay plates using a robotic workstation (Biomek^R^ FX Beckman). Cells were incubated in the presence of the test compounds for 12 hours.

### Image acquisition and processing

Assay plates were read on the BD Pathway™ 855 Bioimager (Becton Dickinson Biosciences, San Jose, California, USA) equipped with a 430/25 nm/470/30 nm ECFP excitation/emission filter, 500/20 nm/535/30 nm EYFP excitation/emission filter and 635/20 nm/695/55 nm DRAQ5 excitation/emission filter. Images for each well were acquired in the three different channels for ECFP, EYFP and DRAQ5 using a 20× dry objective. The plates were exposed for 0.55 ms (Gain 14) to acquire ECFP images, 0.68 ms (Gain 32) for EYFP images and 0.47 ms (Gain 5) to acquire DRAQ5 images. The far red fluorescence intensity of DRAQ5 was used to perform automated segmentation of the cell nuclei and in turn to quantify the total cell number.

### Data analysis

The data output of the BD Pathway Bioimager is as standard text files. These files contained the raw fluorescence data for each cells population. Data were imported into the data analysis software, BD Image Data Explorer, and the ratios of the ECFP positive cells to EYFP positive cells were determined by dividing the number of cyan fluorescence-emitting single cells by the number of yellow fluorescence-emitting single cells in each well. This procedure was repeated for each well. By measuring changes in the ratio between the cyan and yellow signal, the possible pathway-specific cytotoxicity of each compound could be determined. In order to estimate the quality of the HCS assay, the Z' factor was calculated by the equation: Z' = 1 – [(3×std. dev. of positive controls)+(3×std. dev. of negative controls)/(mean of positive controls) - (mean of negative controls)] as described previously [23].
